# Influence of the presence of mannose-binding lectin polymorphisms on the occurrence of leishmaniasis: a systematic review and meta-analysis^☆^

**DOI:** 10.1016/j.abd.2021.08.004

**Published:** 2022-03-21

**Authors:** Wonei de Seixas Vital, Felipe Jules de Araújo Santos, Maurício Leandro Fernandes Gonçalves, Claudia Dantas Comandolli Wyrepkowski, Rajendranath Ramasawmy, Silvania da Conceição Furtado

**Affiliations:** a Pontifícia Universidade Católica do Paraná, Curitiba, PR, Brazil; b Fundação de Medicina Tropical Dr. Heitor Vieira Dourado, Manaus, AM, Brazil; c Universidade Estácio de Sá, Manaus, AM, Brazil; d Instituto Nacional de Pesquisas da Amazônia, Manaus, AM, Brazil; e Department of Morphology, Universidade Federal do Amazonas, Manaus, AM, Brazil

**Keywords:** Leishmaniasis, Mannose-binding lectin, Polymorphism, genetic

## Abstract

**Background:**

Leishmaniasis is caused by an intracellular protozoan of the *Leishmania* genus. Mannose-binding lectin (MBL) is a serum complement protein and recognizes lipoprotein antigens in protozoa and the bacterial plasma membrane. Nucleotide variants in the promoter region and exon 1 of the MBL gene can influence its expression or change its molecular structure.

**Objective:**

To evaluate, through a systematic review, case-control studies of the genetic association of variants in the MBL2 gene and the risk of developing leishmaniasis.

**Methods:**

This review carried out a search in PubMed, Science Direct, Cochrane Library, Scopus and Lilacs databases for case-control publications with six polymorphisms in the mannose-binding Lectin gene. The following strategy was used: P = Patients at risk of leishmaniasis; I = Presence of polymorphisms; C = Absence of polymorphisms; O = Occurrence of leishmaniasis. Four case/control studies consisting of 791 patients with leishmaniasis and 967 healthy subjects (Control) are included in this meta-analysis. The association of variants in the mannose-binding Lectin gene and leishmaniasis under the allelic genetic model, -550 (H*vs*. L), -221 (X *vs*. Y), +4 (Q *vs*. P), CD52 (A *vs*. D), CD54 (A *vs*. B), CD57 (A *vs*. C) and A/O genotype (A vs. O) was evaluated. International Prospective Register of Systematic Reviews (PROSPERO): CRD42020201755.

**Results:**

The meta-analysis results for any allelic genetic model showed no significant association for the variants within the promoter, the untranslated region, and exon 1, as well as for the wild-type A allele and mutant allele O with leishmaniasis.

**Study limitations:**

Caution should be exercised when interpreting these results, as they are based on a few studies, which show divergent results when analyzed separately.

**Conclusions:**

This meta-analysis showed a non-significant association between the rs11003125, rs7096206, rs7095891, rs5030737, rs1800450, and rs1800451 polymorphisms of the Mannose-binding Lectin gene and leishmaniasis in any allelic and heterogeneous evaluation.

## Introduction

Leishmaniasis, a disease spread through bites by infected Phlebotomine sandflies of the genus *Phlebotomus*, is caused by intracellular parasitic protozoa belonging to the *Leishmania* genus.^1^, ^2^ Leishmaniasis exhibits a variety of clinical characteristics, and is classified as cutaneous leishmaniasis (CL), mucocutaneous leishmaniasis (ML), and visceral leishmaniasis (VL).^2^ Twelve million people in 98 countries have leishmaniasis. Approximately 1.5–2.0 million and 500,000 new cases of CL and VL, respectively, are detected annually, and the disease causes about 40,000 deaths a year.^3^ Leishmaniasis is influenced by several factors such as the host genetic origin, nutritional aspects, *Leishmania spp.,* environmental and the immunological aspects.^4^ A recent study reported that genetic variations in the host might play a key role in the susceptibility to leishmaniasis.^5^ Many genes have been investigated, showing a strong relationship between single nucleotide polymorphisms (SNPs) and the risk of developing leishmaniasis, including IFN-G (interferon-gamma)^6^ and IL-6 (interleukin-6).^7^

Mannose-binding lectin (MBL) is a pathogen recognition receptor (PRR) and plays a critical role in host immunity. MBL leads to the activation of the complement system.^8^, ^9^ This oligomeric protein consists of structural subunits formed by three identical 32 kD (kilodaltons) polypeptides, each containing a cross-link with the N-terminal region of cysteine, collagen linked to the neck region, and a region of the C-terminal domain that recognizes carbohydrates in microorganisms.^10^

Across multiple lectin domains, carbohydrates such as mannose (six-carbon carbohydrates) are found on the surface of several pathogens, including *Trypanosoma cruzi*,^11^ *Plasmodium falciparum*,^12^ and *Mycobacterium tuberculosis*.^13^ After the recognition of these molecules by lectin, serine proteases are activated to facilitate opsonization (phagocytosis) by macrophages and lysis of the microorganism surface. ^14^ The infectious form of *Leishmania* (promastigote) is characterized by the presence of lipophosphoglycans (LPG) and other molecules such as mannose.^15^, ^16^ These components act as pathogen-associated molecular patterns (PAMPs) that are recognized by complement components.^17^, ^18^

The MBL2 gene is located on chromosome 10 (10q11.2-q21).^19^ Several SNPs have been identified in this gene, which are known for their functional effect on the development of infectious diseases.^20^, ^21^, ^22^ Functional SNPs, located in the promoter region, such as the -550 H/L SNP (G > C substitution, rs11003125), -221 X/Y (C > G substitution, rs7096206) and +4 Q/P (C > T substitution, rs7095891) located in the untranslated region, can regulate the transcription rate of the gene. ^23^ In the first exon, there are three SNPs located at codon 52 CGT > TGT (rs5030737), codon 54 GGC > GAC (rs1800450) and codon 57 GGA > GAA (rs1800451), corresponding to amino acid changes of arginine to cysteine ​​(Arg52Cys, allele D), glycine to aspartic acid (Gly54Asp, allele B) and glycine to glutamic acid (Gly57Glu, allele C) in the collagen region of the polypeptide chain, respectively.^24^ These three polymorphisms form the AO system, in which the wild-type allele is described as the A allele and the O allele as a mutant. The A/O genotype is correlated with low levels of the protein and is undetectable for the O/O genotype.^25^

Conflicting results are observed between the MBL2 gene variants and susceptibility to leishmaniasis. Variants featuring high levels of the protein have been associated with susceptibility to VL in Africa,^26^ northeastern Brazil,^27^ and India.^28^ However, a study performed in individuals with CL in northern Amazonas, Brazil, showed that all polymorphisms related to low levels of MBL had a strong association with susceptibility.^29^

Some studies have been conducted previously to assess the effects of the MBL2 gene polymorphisms on the evolution of the infection in leishmaniasis, with contradictory results due to the small sample size, which lacks adequate power to detect the effects of MBL2 gene polymorphisms on leishmaniasis.

To date, no systematic review has been performed on MBL2 gene variants and leishmaniasis. The use of a meta-analysis as a statistical tool to explore risk factors associated with different genetic diseases can provide a reliable conclusion. This systematic review included case-control studies of the genetic association of variants (rs11003125, rs7096206, rs7095891, rs5030737, rs1800450, and rs1800451) in the MBL2 gene and the risk of developing leishmaniasis. This systematic review is in the International Prospective Register of Systematic Reviews (PROSPERO): CRD42020201755.

## Materials and methods

### Database search

This systematic review was carried out in accordance with the recommendations of the PRISMA (Preferred Reporting Items for Systematic Reviews and Meta-Analyses) protocol. ^30^ The PubMed, Science Direct, Cochrane Library, Scopus, and Lilacs databases were searched up to December 2019 by three independent reviewers, with no language or time restrictions.

The following strategy was used: P = Patients at risk for leishmaniasis; I = Presence of polymorphisms; C = Absence of polymorphisms; O = Occurrence of leishmaniasis. The following terms were used in the search: *(“Leishmaniasis ”OR “Cutaneous Leishmaniasis” OR “Visceral Leishmaniasis “OR “Leishmania Infection” OR “Leishmania Infections”) AND (“Mannose-binding Lectin” OR “Mannose-binding Lectin 2” OR “MBL” OR “MBL2”) AND (“Polymorphism” OR “Polymorphisms” OR “Single Nucleotide Polymorphism” OR “Single Nucleotide Polymorphisms”)*. The references cited in eligible articles were manually searched to identify additional publications. Ethical approval and informed consent were not required as this study was based on previously published studies, and there was no direct patient contact or influence on patient care.

### Study selection

Two researchers independently evaluated all search results. The inclusion criteria were as follows: 1) Case-control study, 2) rs11003125 (-550), rs7096206 (-221), rs7095891 (+4), rs5030737 (Codon 52), rs1800450 (CD54) and rs1800451 (CD57) polymorphisms, 3) Studies with sufficiently available genotyping data to calculate the Odds Ratios (OR) with 95% Confidence Intervals (95% CI) and 4) the Hardy-Weinberg Equilibrium (HWE). The exclusion criteria were: 1) Non-case-control study, 2) Case reports, 3) Reviews, 4) Animal studies, 5) Editorials, 6) Studies with no available data, 7) Studies with meta-analysis, 8) Other polymorphisms and 9) Duplicate data. Subsequently, all selected articles were verified by a third researcher, who resolved the divergences.

### Data extraction

Two researchers independently extracted the following data from the included studies: year of publication, first author, study region, ethnic group, clinical form, number of samples, age, and studied SNPs. Disagreements between the researchers were discussed and resolved by consulting a third researcher.

### Quality score evaluation

The Newcastle-Ottawa scale (Table 1) was used to assess the quality of the eligible studies. Using this system, each included study was submitted to three judgments: 1) Selection of study groups; 2) Comparability of the groups and 3) Outcome of interest (Case-Control). Three researchers independently calculated the score for each publication. The scores ranged from 0 to 9. Studies with a score > 6 were considered of high quality, while those with a score < 6 were listed as of low quality. Disagreements between the researchers were discussed in the group and resolved by consensus.

**Table 1:** Newcastle-Ottawa scale of included studies.

Study	Selection				Comparability			Outcome	
	Representativeness	Selection of the non-exposed cohort	Investigation	Final score not present at the start	Comparability (confounding)	Evaluation of outcomes	Duration/Screening	Monitoring of adequacy	Total
Felipe FJ	*	*	*	*	**	*	*	*	9
Salsabil H	*	*	*	*	**	*	*	*	9
Alonso DP	*	*	*	*	*	*	*	*	8
Anshuman M	*	*	*	*	*	*	*	*	8

Each item was graded with a maximum score of one point (one *), with the exception of comparability, which allowed for two points.

### Statistical analysis

The meta-analysis evaluated the association of the MBL2 gene and leishmaniasis under the allelic genetic model, -550 (H *vs*. L), -221 (X *vs*. Y), +4 (Q *vs*. P), CD52 (A *vs*. D), CD54 (A *vs*. B), CD57 (A *vs*. C) and the A/O genotype (A *vs*. O). I^2^ was used to assess the heterogeneity between studies, where the values ​​25%, 50% and 75% corresponded to low, moderate, and high heterogeneity, respectively. The fixed model was used when I^2^ < 50%, and the random model was used when I^2^ > 50%. Pooled ORs were calculated using the Mantel-Haenszel method, and the statistical significance of OR was determined using Z statistics. In both models, p = 0.005 was considered statistically significant. The RStudio software (www.rstudio.com/products/rstudio/), version 1.3.1 for Windows was used for the statistical analysis of the study. Packages (“tidyverse”), (“meta”), (“metafor”).

## Results

### Characteristics of the included studies

**Fig. 1:**
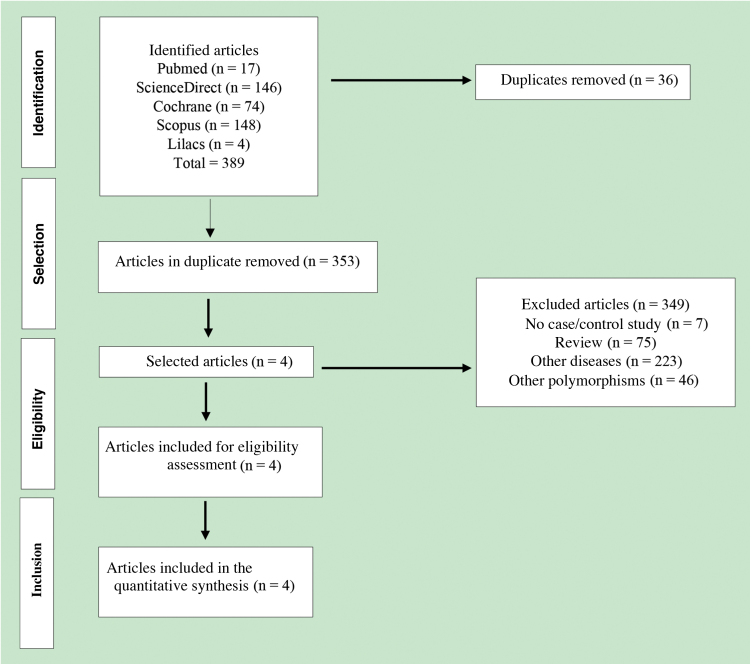
Flowchart of the literature review process according to the PRISMA protocol.

A total of 389 published articles were identified using scientific literature databases (Fig. 1). Among the selected articles, 35 were removed due to duplication, 349 articles were excluded for not meeting the inclusion criteria. Finally, only four articles,^26^, ^27^, ^28^, ^29^ that met the mandatory criteria, were included in the meta-analysis. These studies were published in English between the years 2007 and 2015 (Table 2).

**Table 2:** Leishmaniasis. Characteristics of the studies included in the systematic review.

Year	Study	Country/Region	Ethnic group	Clinical forms	Case Control samples		Age, mean years ± SD or mean (range) Case Control		SNPs
2007	Alonso DP	Brazil/Northeast	Mixed	VL	61	231	6 months to 73 years	6 months to 73 years	rs11003125, rs7096206, rs5030737, rs1800450, rs1800451
2015	De Araújo FJ	Brazil/ North	Mixed	CL	400	382	311 men (32 ± 15.5 years)	225 men (38 ± 17.6 years)	rs11003125, rs7096206, rs7095891, rs5030737, rs1800450, rs1800451
							89 women (32 ± 13.7)	157 women (34 ± 17.5)	
2013	Salsabil H	Morocco/North	African	VL	112	139	7 ± 12 years	8.5 ± 12 years	rs7096206, rs1800450, rs1800451
2015	Anshuman M	India	NR	VL	218	215	28,7 ± 16.7 years	35.3 ± 16.2 years	rs7095891

NR, Not Reported.

One study was conducted on African children.^26^ Two other studies were carried out in mixed populations from the northeastern region of Brazil, consisting of 21% European, 31% African, and 48% Native American descendants,^27^ and from the northern region of Brazil (state of Amazonas), with a mixed population of 10% African, 40% European, and 50% Native American descendants.^29^ The fourth study was conducted in India but did not specify the ethnicity of the studied subjects.^28^ Three studies analyzed patients with VL,^26^, ^27^, ^28^ and one analyzed patients with CL.^29^ The identified *Leishmania spp* were: *L. chagasi,* ^27^ *L. infantum*,^26^ *L. guyanensis*^29^ and *L. donovani*.^28^ One study analyzed all polymorphisms targeted by this meta-analysis,^29^ while another analyzed only five SNPs (rs11003125, rs7096206, rs5030737, rs1800450 e rs1800451).^27^ One study analyzed three SNPs (rs7096206, rs1800450, and rs1800451),^26^ and the remaining study analyzed only one SNP (rs7095891).^28^ Two studies provided sufficient data to perform the A/O system analysis.^27^, ^29^ A total of 1758 individuals participated in these studies (791 patients and 967 controls). According to the Newcastle-Ottawa scale, two studies scored 9 points, and two scored 8 points (Table 1). The frequency of genotypes and alleles are organized in Table 3.

**Table 3:** Allelic genetic model adopted in the meta-analysis to evaluate the association of MBL2 gene polymorphisms and leishmaniasis.

Study	Year	Total sample		Case					Control				
**-550**		**Cases**	**Control**	**HH**	**HL**	**LL**	**H**	**L**	**HH**	**HL**	**LL**	**H**	**L**
Alonso DP	2007	60	226	4 (7)	29 (48)	27 (45)	37 (31)	83 (69)	25 (11)	99 (44)	102 (45)	149 (33)	303 (67)
de Araújo FJ	2015	365	332	49 (13)	167 (46)	149 (41)	265 (36)	465 (64)	53 (16)	147 (44)	132 (40)	253 (38)	411 (62)
**-221**				**XX**	**XY**	**YY**	**X**	**Y**	**XX**	**XY**	**YY**	**X**	**Y**
Alonso DP	2007	60	226	0 (0)	14 (23)	46 (77)	14 (12)	106 (88)	4 (2)	64 (28)	158 (70)	72 (16)	380 (84)
Salsabiln H	2013	112	139	15 (13)	39 (34)	58 (52)	69 (31)	155 (69)	20 (14)	71 (51)	48 (35)	111 (40)	167 (60)
de Araújo FJ	2015	365	332	30 (08)	125 (34)	210 (58)	185 (25)	545 (75)	12 (4)	77 (23)	243 (73)	101 (15)	563 (85)
**+4**				**QQ**	**QP**	**PP**	**Q**	**P**	**QQ**	**QP**	**PP**	**Q**	**P**
de Araújo FJ	2015	365	332	17 (5)	95 (26)	253 (69)	129 (18)	601 (82)	9 (3)	94 (28)	229 (69)	111 (17)	551 (83)
Anshuman M	2015	218	215	12 (6)	72 (33)	134 (61)	96 (22)	340 (78)	22 (10)	83 (39)	110 (51)	127 (29)	303 (71)
**CD52**				**AA**	**AD**	**DD**	**A**	**D**	**AA**	**AD**	**DD**	**A**	**D**
Alonso DP	2007	61	231	58 (95)	3 (5)	0 (0)	119 (98)	3 (2)	218 (94)	12 (5.1)	1 (0.9)	448 (97)	14 (3)
de Araújo FJ	2015	366	332	342 (93)	22 (6)	2 (1)	706 (96)	26 (04)	306 (92)	26 (8)	0 (0)	638 (96)	26 (4)
**CD54**				**AA**	**AB**	**BB**	**A**	**B**	**AA**	**AB**	**BB**	**A**	**B**
Alonso DP	2007	61	231	41 (67)	19 (31)	1 (2)	101 (83)	21 (17)	117 (51)	96 (41)	18 (8)	330 (71)	132 (29)
Salsabiln H	2013	104	133	71 (68)	27 (26)	6 (6)	169 (81)	39 (19)	96 (72)	32 (24)	5 (4)	224 (84)	42 (16)
de Araújo FJ	2015	366	332	215 (59)	121 (33)	30 (8)	551 (75)	181 (25)	211 (63)	105 (32)	16 (5)	527 (79)	137 (21)
**CD57**				**AA**	**AC**	**CC**	**A**	**C**	**AA**	**AC**	**CC**	**A**	**C**
Alonso DP	2007	61	231	55 (90)	6 (10)	0 (0)	116 (95)	6 (5)	202 (87)	28 (12)	1 (1)	432 (94)	30 (6)
Salsabiln H	2013	104	133	88 (85)	15 (14)	1 (1)	176 (91)	17 (9)	111 (83)	18 (14)	4 (3)	240 (90)	26 (10)
de Araújo FJ	2015	365	332	255 (70)	91 (25)	19 (5)	601 (82)	129 (18)	270 (81)	57 (17)	5 (2)	597 (90)	67 (10)
**A/O**				**AA**	**AO**	**OO**	**A**	**O**	**AA**	**AO**	**OO**	**A**	**O**
Alonso DP	2007	61	231	36 (59)	20 (33)	5 (8)	92 (75)	30 (25)	95 (41)	103 (45)	33 (14)	293 (63)	169 (37)
de Araújo FJ	2015	365	332	126 (35)	155 (42)	84 (23)	407 (56)	323 (44)	153 (46)	133 (40)	46 (14)	439 (66)	225 (34)

### Meta-analysis

**Fig. 2:**
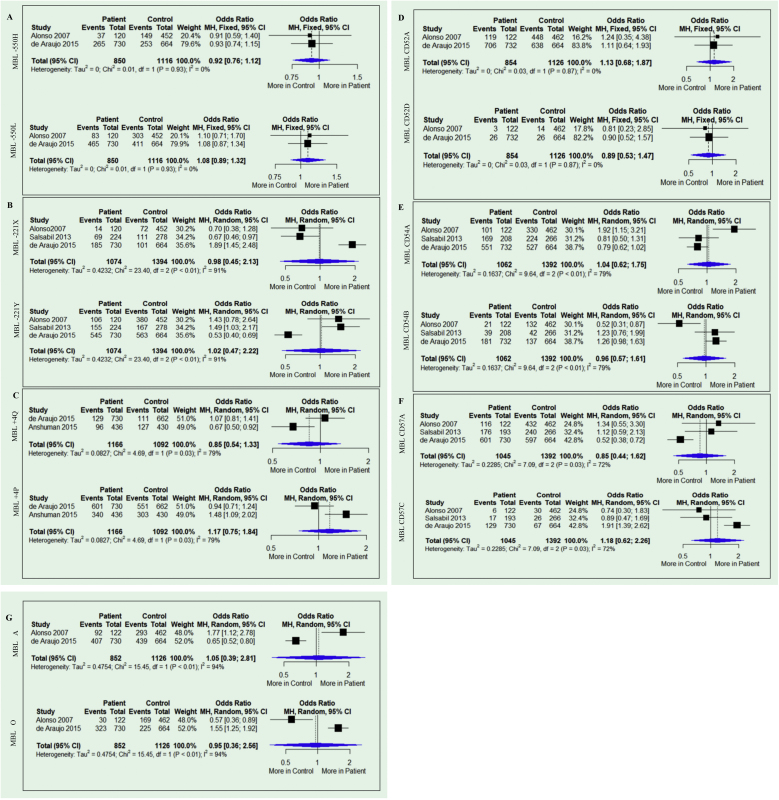
Forest plot of the meta-analysis of the comparison between mutant alleles *versus* wild-type alleles of the SNPs.

The results of the meta-analysis are shown in Fig. 2. None of the analyses for any allele genetic model for the two variants (-550 and -221) in the promoter and the +4 variants in the untranslated regions showed any association with susceptibility or resistance to Leishmaniasis (−550H Allele: OR = 0.92; 95% CI = 0.76–1.12; p = 0.93; I^2^ = 0% and −550 L allele: OR = 1.08; 95% CI = 0.89–1.32; p = 0.93; I^2^ = 0%), -221X allele: OR = 0.98; 95% CI = 0.45–2.13; p = 0.01; I^2^ = 91% and -221Y allele: OR = 1.02; 95% CI = 0.47–2.22; p = 0.01; I^2^ = 91%) and Q + 4 allele: OR = 0.85; 95% CI = 0.54–1.33; p = 0.03; I^2^ = 79% and P allele: OR = 1.17; 95% CI = 0.75–1.84; p = 0.03; I^2^ = 79%). Similar results were obtained for variants located on exon 1 CD52 (allele A of the allele: OR = 1.13; 95% CI = 0.68–1.87; p = 0.87; I^2^ = 0% and allele D of the allele: OR = 0.89; 95% CI = 0.53–1.47; p = 0.87; I^2^ = 0%), CD54 (allele A: OR = 1.04; 95% CI = 0.62–1.75; p = 0.01; I^2^ = 79% and allele B: OR = 0.96; 95% CI = 0.57–1.61; p = 0.01; I^2^ = 79%) and CD57 ( allele A: OR = 0.85; 95% CI = 0.44–1.62; p = 0.03; I^2^ = 72% and allele C: OR = 1.18; 95% CI = 0.62–2.26; p = 0.03; I^2^ = 72%). The presence of the wild-type A allele (A allele: OR = 1.05; 95% CI = 0.39–2.81; p = 0.01; I^2^ = 94%) and the mutant O allele (O allele: OR = 0.95; 95% CI = 0.36–2.56; p = 0.01; I^2^ = 94%) were also not associated with susceptibility or resistance.

## Discussion

MBL recognizes the presence of mannose on the surface of pathogens to promote opsonization and activation of the complement system.^31^ MBL plays a key role in the innate immune response,^32^ highlighting its serum concentration as a requirement for predisposition to the development of human infectious diseases.^33^, ^34^ MBL2 gene variants have been associated with an increased risk of infections caused by protozoa.^35^, ^36^ However, few studies have investigated genetic variants in the MBL2 gene in Leishmaniasis.^26^, ^27^, ^28^, ^29^ Three studies have suggested that variants correlated with low circulating levels of MBL are protective for VL,^26^, ^28^ while one study showed susceptibility to CL.^29^

The conflicting results generated by most studies had weak statistical power due to the small sample size included. To clarify conflicting results in genetic association studies, a meta-analysis offers a powerful method to synthesize data obtained from independent studies.^37^ To address the limitations of case-control studies, the present meta-analysis was performed to provide statistical evidence of the association between MBL2 gene polymorphisms and susceptibility to leishmaniasis with clustered ORs. To date, this is the first meta-analysis to address the association between the described polymorphisms and leishmaniasis. Previous meta-analyses have suggested an association of polymorphisms in the IL2RA (Interleukin 2 Receptor alpha)^38^ and SLC11A1 (solute carrier family 11 member 1)^39^ genes with the clinical aspects of leishmaniasis.

In the present study, data from four studies were analyzed according to the low- and high- MBL producing alleles. However, the meta-analysis analyses showed no association between MBL2 gene alleles and susceptibility to leishmaniasis (Fig. 2). High heterogenicity was observed for the variants: -550 H/L (91%), +4 Q/P (79%), CD54 A/B (79%), CD57 A/C (72%) and A/O (94%). This can be explained mainly by the ethnic miscegenation of the individuals included in the selected studies. Three studies investigated patients with VL,^26^, ^27^, ^28^ and one patient was investigated with CL.^29^ In each study, the species of the etiologic agent were different. It is important to note that the heterogeneity value influences the adequate statistical model. Studies with small sample sizes can show unreliable results. As a consequence, the random model must always be applied.^40^ Among the selected studies, one analyzed all six target polymorphisms, the diplotypes, and also the haplotypes,^29^ with high sample size.

However, caution should be exercised when interpreting these results, as they are based on a few studies, which show divergent results when analyzed separately. Therefore, further studies are needed to confirm whether the variants that determine low serum levels are susceptible or protective. The great importance of the association study involving genetic markers in leishmaniasis is emphasized, aiming at a new understandings of the molecular mechanisms of the disease. The variants can be used as molecular markers of the individual predisposition to certain types of diseases or as therapeutic targets in the development of new drugs.

## Conclusion

Overall, this meta-analysis showed no significant association between polymorphisms rs11003125, rs7096206, rs7095891, rs5030737, rs1800450, and rs1800451 of the MBL2 gene and leishmaniasis.

## Financial support

None declared.

## Conflicts of interest

None declared.

## Authors' contributions

Wonei de Seixas Vital: Conceptualization, Formal analysis, Methodology, Supervision, Validation, Writing – review & editing.

Felipe Jules de Araújo Santos: Conceptualization, Formal analysis, Methodology, Supervision, Validation, Writing – review & editing.

Maurício Leandro Fernandes Gonçalves: Data curation, Formal analysis, Methodology, Writing – original draft.

Claudia Dantas Comandolli Wyrepkowski: Data curation, Formal analysis, Methodology.

Rajendranath Ramasawmy: Data curation, Formal analysis, Methodology, Writing – review & editing.

Silvania da Conceição Furtado: Conceptualization, Formal analysis, Methodology, Supervision, Validation, Writing – review & editing.
